# Characteristics of Pregnancy Course in an Infant With Cerebral Palsy Showing Decreased Fetal Movement

**DOI:** 10.1155/ogi/1079227

**Published:** 2026-01-29

**Authors:** Shiho Nagayama, Hironori Takahashi, Hanako Ohtachi, Manabu Ogoyama, Rie Usui, Hirotada Suzuki, Yoshio Matsuda, Hiroyuki Fujiwara

**Affiliations:** ^1^ Department of Obstetrics and Gynecology, Jichi Medical University, Shimotsuke, Tochigi, Japan, jichi.ac.jp; ^2^ Department of Obstetrics, Toho Women’s Clinic, Tokyo, Japan

**Keywords:** cerebral palsy, DFM, fetomaternal hemorrhage, placental abruption, velamentous insertion

## Abstract

**Objective:**

To clarify the characteristics of patients with cerebral palsy (CP) showing decreased fetal movement (DFM).

**Methods:**

Among patients with CP between January 2009 and February 2021, we collected cases of DFM from the causal analysis report. We retrieved the clinical course and the causes of CP.

**Results:**

Of 2834 cases of CP, 225 (8%) patients were included in this study. Some form of hypoxia was the most common cause (117 cases, 52%) followed by placental abruption (45 cases, 20%) and fetomaternal hemorrhage (FMH) (32 cases, 14%). The duration from DFM to delivery was longer in cases of FMH than in placental abruption (*p* < 0.001). The duration less than 6 h was only observed in one (4%) case of FMH, whereas it was observed in 32 (73%) cases of placental abruption. In contrast, the cases with durations of more than 24 h accounted for 36% (10/28) in FMH cases. Next, we focused on hypoxia cases. Marginal or velamentous insertion accounted for 21% (22/106) of the hypoxia cases. Umbilical artery pH and base excess were worse in cases of normal site insertion than those of marginal or velamentous insertion.

**Conclusion:**

DFM was seen in 8% of patients with CP. FMH required more time from the DFMs to delivery than cases of placental abruption. Fetuses with a velamentous or marginal cord insertion may have a risk of CP.

## 1. Introduction

Cerebral palsy (CP) is a neurological disorder that appears during infancy or early childhood. It permanently affects body movement and muscle coordination. The prevalence of CP is 2.11 per 1000 live births [1]. The causative events usually occur during the perinatal period and early infancy less than 4 weeks. The risk factors include premature birth, obstetrical diseases including placental abruption and fetomaternal hemorrhage (FMH), persistent hypoxia, acidosis, and infections.

A decreased fetal movement (DFM) is a sign of deteriorating fetal well‐being. Some infants who later develop CP exhibit DFM. Although there are only a few reports examining the extent to which DFM is related to CP, a single‐center retrospective study demonstrated that DFM was associated with pediatric hospitalizations including infants with CP [2]. Zamstein et al. [2] compared 439 women who felt DFM from the second to third trimester with 240,000 control pregnant women. The number of infants with DFM significantly increased among those hospitalized for neurological causes (hazard ratio: 1.54, 95% CI: 1.0–2.37, *p* = 0.048). Thus, DFM increases the risk of CP. However, the duration from DFM to delivery remains unknown in patients with CP. There are several diseases that can induce CP. Are there any differences in the clinical characteristics depending on the disease? Thus, we examined the characteristics of patients with CP who exhibited DFM using a database from the Japan Obstetric Compensation System for Cerebral Palsy (JOCSC).

## 2. Materials and Methods

This study was approved by the Institutional Review Board of our institution (approval number: rindai 21‐023) and the Review Board of JOCSC (approval number: rinjinsho 2021‐02). We did not obtain written informed consent from participating women. Because of the retrospective design, the ethics committee did not require informed consent from each patient. Information on how to opt out if desired was shown on a freely accessible website (https://www.sanka-hp.jcqhc.or.jp/documents/study_notice/index.html).

To support the financial burden for children with CP and their family, JOCSC was established in 2009 as the nationwide no‐fault compensation system (https://www.sanka-hp.jcqhc.or.jp/) [3–5]. The candidate CP cases were reviewed by a review committee including obstetricians, pediatricians, midwives, and lawyers. If the candidate patient is judged to be eligible to receive compensation by this review committee, the causes for CP are analyzed individually by the causal analysis committee consisting of obstetricians, pediatricians, midwives, and lawyers. The committee discussed the possible cause of CP, and their discussion was described in the causal analysis report. Children eligible for the JOCSC were described previously (https://www.mhlw.go.jp/stf/seisakunitsuite/bunya/kenkou_iryou/iryou/i-anzen/sanka-iryou/index.html) [5, 6].

**Figure 1:**
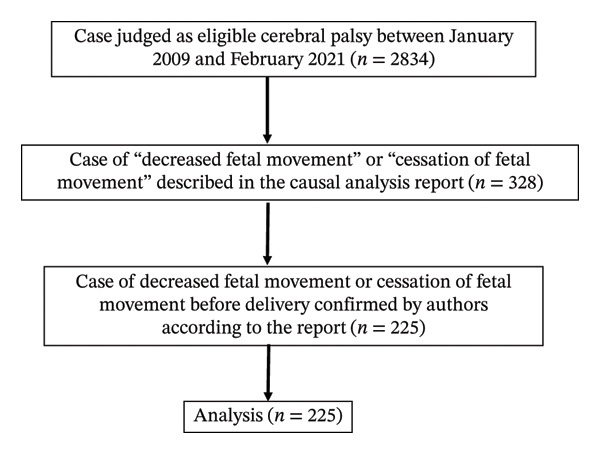
Selection criteria for target patients.

Briefly, the eligible cases were that of severe CP associated with each reason during perinatal period at delivery, not caused by congenital diseases, or by factors during the neonatal period. The patients need to have a CP equivalent to a level 1 or 2 physical disability certificate. The detailed inclusion criteria were changed yearly. Patients born at ≥ 33 weeks and birth weight of ≥ 2000 g or at ≥ 28 weeks with apparent asphyxia at birth between January 1, 2009, and December 31, 2014, were candidates. From January 1, 2015, until December 31, 2021, patients born at ≥ 32 weeks and birth weight of ≥ 1400 g or at ≥ 28 weeks with apparent asphyxia at birth for children were candidates. From January 1, 2022, patients born at ≥ 28 weeks were candidates. At the start in 2009, each pregnant woman paid a premium of 30,000 yen (approximately USD 200). However, the number of children who was applied in this insurance was smaller than expected, resulting in a financial surplus. To reduce this surplus, the system gradually expanded its coverage by making the eligibility criteria less strict. Consequently, the types of cases covered have changed from year to year, and the characteristics of the included patients have slightly differed across fiscal years. Applications for the compensation are accepted from the child’s first birthday until fifth birthday, and 99.9% of delivery facilities in Japan are members of this compensation system. The database preserves the confidentiality. The age and name of pregnant women, their detailed obstetric history, their medical and social complications, or managed hospitals are not disclosed. We collected cases with “DFM” and “cessation of fetal movement” during the observation period from the database (data masking version) described in Results between January 2009 and February 2021 (Figure 1). Some of the investigators (Shiho Nagayama, Hironori Takahashi, and Hanako Ohtachi) retrieved records of each case from the causal analysis reports. The retrieved items were as follows: primiparous or multiparous, the mode of pregnancy, singleton or multifetal pregnancy, smoking status, the presence or absence of preeclampsia, the presence or absence of gestational diabetes mellitus, mode of delivery, delivery at gestational week, birth weight, Apgar score at 1 and 5 min, data of umbilical artery (pH and base excess), the presence or absence of meconium staining, placental weight, the cord insertion site, the presence or absence of true knot, and duration from DFM to delivery. Regarding placental abruption and FMH, we confirmed the cases that did not show respiratory acidosis. The causes of CP were categorized by investigators (Shiho Nagayama, Hironori Takahashi, and Hanako Ohtachi) based on the review board’s opinion of JOCSC as follows: placental abruption, hypoxia, infection, uterine rupture, umbilical cord prolapse, FMH, twin‐to‐twin transfusion syndrome including circulatory imbalance in monozygotic twins, cerebral hemorrhage or infarction, postnatal event (e.g., late‐onset Group B streptococcal infection and nuclear icterus), others, and unknown. The hypoxia group was defined as follows: the review board determined that hypoxic event occurred; however, the direct cause could not be confirmed. For example, an interruption of the cord blood with some reasons was included in this group. Statistical analyses were performed using the statistical software package JMP Pro Version 17 for Macintosh (SAS Institute, Cary, NC, USA). The Wilcoxon sum‐rank test and Fisher’s exact test (two‐tailed) were used to compare the two groups. A *p* value < 0.05 was considered significant.

## 3. Results

A total of 2834 cases were judged as eligible CP (Figure 1). Of those, we extracted 328 cases of “DFM” and “cessation of fetal movement” from the database of the causal analysis reports. Of these cases, we checked each case and selected pregnant women with “decreased or deceased fetal movement” antepartum or intrapartum. Finally, 225 (8%) patients were included in this study (Figure 1). The background is shown in Table 1. The hypoxia judged by the committee was the most common cause of CP (52%), followed by placental abruption (20%) and FMH (14%). At admission, reassuring fetal heart rate patterns were present in 51% of the cases. Regarding the duration from DFM to delivery, the most frequent duration was 6–24 h (46%).

**Table 1:** Background of patients with cerebral palsy infant manifesting decreased fetal movement (*n* = 225).

Primiparous, *n* (%)	144 (64)
ART pregnancy, *n* (%)	15 (7)
Current smoker, *n* (%)	9 (4)
Illicit drug use, *n* (%)	0 (0)
GDM, *n* (%)	3 (1)
Preeclampsia, *n* (%)	11 (5)
Singleton, *n* (%)	219 (97)
Cause of cerebral palsy, *n* (%)	
Hypoxia	117 (52)
Placental abruption	45 (20)
Fetomaternal hemorrhage	32 (14)
Infection	9 (4)
Cerebral bleeding or infarction	6 (3)
Twin‐twin transfusion syndrome	5 (2)
Unknown	11 (5)
Reassuring pattern at admission, *n* (%)	115 (51)
Duration from decreased fetal movement to delivery (hours), *n* (%)	
< 2	17 (17/202: 8)
2–6	35 (35/202: 17)
6–24	92 (92/202: 46)
24–48	17 (17/202: 8)
> 48	41 (41/202: 20)
NA	23

Abbreviations: ART, assisted reproductive technology; GDM, gestational diabetes mellitus; NA, not available.

Delivery information is shown in Table 2. Cesarean section (CS) was required in 195 (87%) cases. Deliveries at term and late preterm (34 + 0–36 + 6 weeks) comprised 112 (50%) and 63 (28%) cases, respectively. The median (interquartile range: IQR) birth weight was 2394 g. The median umbilical artery pH was 7.10. Cases with pH less than 7.0 accounted for 31%, and those with pH more than 7.2 accounted for 37%. Regarding the cord insertion, marginal or velamentous insertion accounted for approximately 15% (30/200) of the cases, and meconium staining was observed in 42%.

**Table 2:** Delivery outcome (*n* = 225).

Delivery mode, *n* (%)	
CS	195 (87)
Vaginal	30 (13)
GA at delivery (week), median (IQR)	258 (240–273)
28 + 0–29 + 6	14
30 + 0–31 + 6	12
32 + 0–33 + 6	24
34 + 0–36 + 6	63
37 + 0–41 + 6	112
Birth weight (g), median (IQR)	2394 (1920–2841)
< 1000	2
1000–1499	26
1500–1999	36
2000–2499	62
2500–2999	52
3000–3499	38
≥ 3500	9
Apgar score at 1 min, median (IQR)	1 (1–3)
0	48
1–3	125
4–6	35
7–10	16
NA	1
Apgar score at 5 min, median (IQR)	4 (2–6)
0	25
1–3	70
4–6	83
7–10	46
NA	1
Umbilical artery pH, median (IQR)	7.10 (6.94–7.31)
7.2 ≤ pH	75
7.1 ≤ pH < 7.2	24
7.0 ≤ pH < 7.1	39
6.9 ≤ pH < 7.0	17
6.8 ≤ pH < 6.9	12
6.7 ≤ pH < 6.8	14
pH < 6.7	20
NA	24
Base excess, median (IQR)	−11.8 (−17.9–−4.6)
−5 ≤ BE	53
−10 ≤ BE < −5	28
−15 ≤ BE < −10	41
−20 ≤ BE < −15	31
−25 ≤ BE < −20	15
BE < −25	21
NA	36
Placental weight (g), median (IQR)	500 (426–580)
Cord insertion, *n* (%)	
Normal site	170 (75)
Marginal	22 (10)
Velamentous	8 (4)
NA	25 (11)
Hypercoiling, *n* (%)	22 (10)
True knot of umbilical cord, *n* (%)	11 (5)
Meconium staining, *n* (%)	95 (42)

Abbreviations: BE, base excess; CS, cesarean section; GA, gestational age at delivery; IQR, interquartile range; NA, not available.

Although hypoxia is the most common group of CP in this study, the specific pathogenesis cannot be determined or unified in cases with the hypoxia group. Thus, we compared clinical findings of placental abruption with those of FMH (Table 3). Regarding delivery week, placental abruption occurred significantly earlier than FMH (*p* = 0.024). Nonreassuring fetal status including loss of variability and/or prolonged deceleration was detected in all the case but one in FMH. Umbilical artery pH (*p* = 0.001) and base excess (*p* < 0.001) were worse in cases of placental abruption than those of FMH. There were no cases of respiratory acidosis in the two groups (data not shown). Regarding the duration from DFM to delivery, the duration for FMH was longer than that for placental abruption (*p* < 0.001). The duration less than 6 h was only observed in one (4%) case of FMH and in 32 (73%) cases of placental abruption. In addition, a duration of more than 24 h accounted for 36% in FMH cases.

**Table 3:** Perinatal characteristics and outcome by causative disease.

	Placental abruption (*n* = 45)	Fetomaternal hemorrhage (*n* = 32)	*p* value
Primiparous, *n* (%)	26 (58)	18 (56)	0.296
ART, *n* (%)	3 (7)	3 (9)	0.662
Preeclampsia, *n* (%)	4 (9)	0 (0)	0.137
Duration from decreased movement to delivery (hours), *n* (%)			< 0.001
< 6	32 (73)	1 (4)	
6–24	9 (20)	17 (61)	
> 24	3 (7)	10 (36)	
NA	1	4	
GA at delivery (week), median (IQR)	36.6 (34.9–38.1)	38.4 (35.9–39.9)	0.024
LOV and/or prolonged deceleration detected, *n* (%)	39/39 (100)^∗^	31/32 (97)^∗^	0.451
Delivery mode, *n* (%)			0.660
CS	39 (87)	30 (94)	
Vaginal delivery	6 (13)	2 (6)	
Birth weight (g), median (IQR)	2374 (1892–2825)	2906.5 (2403.5–3324.5)	0.002
Umbilical artery pH, median (IQR)	6.74 (6.62–6.97)	7.01 (6.88–7.10)	0.001
Base excess, median (IQR)	−23.3 (−28.2–−17.8)	−15.3 (−18.9–−9.1)	< 0.001
Meconium staining, *n* (%)	13 (33)	17 (53)	0.186
Placental weight (g), median (IQR)	475 (414–535)	645 (550–755)	< 0.001

Abbreviations: ART, assisted reproductive technology; CS, cesarean section; GA, gestational age; IQR, interquartile range; LOV, loss of variability; NA, not available.

^∗^Limited to cases in which cardiotocogram was collected for a certain duration.

Next, we focused on hypoxia group. Multiple pathogeneses were included in hypoxia group (Table 4). The median birth weight was 2374 g. Median umbilical artery pH was 7.20, and median base excess was −7. Although cord factors, such as interruption of the blood flow and embolism, may be one of the main causes of hypoxia, cases with marginal or velamentous insertion accounted for 21% (22/106) in this group (Table 4). We compared normal site insertion with marginal or velamentous insertion. Findings of loss of variability or prolonged deceleration during delivery did not differ significantly between the two groups. Umbilical artery pH (*p* = 0.002) and base excess (*p* = 0.015) were worse in cases of normal site insertion than those of marginal or velamentous insertion.

**Table 4:** Perinatal characteristics between central insertion and marginal or velamentous insertion in hypoxia cases.

	All cases (*n* = 117)	Normal site (*n* = 84)	Marginal or velamentous (*n* = 22)	*p* value^∗^
ART pregnancy, *n* (%)	8 (8/115: 7)	6 (7)	1 (5)	0.886
GA at delivery (week), median (IQR)	261 (240–273.5)	262 (239.5–273.75)	250 (238.25–264.75)	0.121
LOV detected during delivery, *n* (%)	90 (77)	64 (76)	17 (77)	1
Prolonged deceleration detected during delivery, *n* (%)	25 (21)	22 (26)	2 (9)	0.150
Delivery mode, *n* (%)				0.267
CS	103 (88)	76 (90)	18 (82)	
Vaginal delivery	14 (12)	8 (10)	4 (18)	
Birth weight (g), median (IQR)	2374 (1959–2753)	2429.5 (1953–2805)	2068 (1809–2403)	0.068
Umbilical artery pH, median (IQR)	7.20 (7.04–7.35)	7.16 (7.03–7.33)	7.35 (7.23–7.39)	0.002
NA	12	8	2	
Base excess, median (IQR)	−7 (‐13–−2)	−8.75 (−13.55–−3.65)	−2.15 (−7.08–−1.98)	0.015
Meconium staining, *n* (%)	54 (46)	44 (52)	7 (32)	0.098
Placental weight (g), median (IQR)	482.5 (430–550)	486 (430–550)	480 (427.75–517.5)	0.726
Duration from decreased fetal movement to delivery (hours), *n* (%)				0.368
< 6	15 (15/104: 14)	11 (11/74: 15)	3 (3/19: 16)	
6–24	53 (53/104: 51)	35 (35/74: 47)	12 (12/19: 63)	
> 24	36 (36/104: 35)	28 (28/74: 38)	4 (4/19: 21)	
NA	13	10	3	
Cord insertion (*n*)				
Central	84 (84/106: 79)			
Marginal or velamentous	22 (22/106: 21)			
NA	11			

Abbreviations: ART, assisted reproductive technology; CS, cesarean section; GA, gestational age; IQR, interquartile range; LOV, loss of variability; NA, not available.

^∗^Compared between normal insertion and marginal or velamentous insertion.

## 4. Discussion

This study generated several findings. DFM was seen in 8% of patients with CP and was mainly associated with some form of hypoxia, placental abruption, or FMH, which accounted for more than 75% of CP cases. FMH required more time from the DFMs to delivery than placental abruption. Although the cause of the hypoxia was not confirmed, DFMs were the common symptom in the hypoxia group. In addition, 20% of cases diagnosed with hypoxia were complicated by marginal or velamentous cord insertion. Patients with normal site insertion were more likely to develop metabolic acidosis than those with marginal or velamentous insertion at birth, although the duration (from DFM to delivery) was not significant between the two groups.

The duration from DFM to delivery was longer for FMH cases than for placental abruption cases. In our study, 73% of abruption cases were delivered within 6 h from DFM to delivery, whereas FMH cases accounted for only 4%. Although FMH patients are less likely to exhibit clinical symptoms, DFM is the most common symptom, occurring in 27%–43% of FMH cases [7, 8]. The absence of accompanying symptoms, including abdominal pain and bleeding, may lead to the delayed delivery. Furthermore, as DFM in FMH cases is due to acidemia following blood transfer from fetus to mother and it was generally slow, the changes are less noticeable. On the other hand, the majority of placental abruption cases involve other symptoms including bleeding and abdominal pain, which may lead to early consultation. Nevertheless, placental abruption can frequently induce CP [9]. In a retrospective study on placental abruption, 43 of 222 abruption cases (19%) had an umbilical artery pH < 7.0 [10], and 20% of the survivors developed CP [11]. Indeed, even if immediate delivery following DFM was performed in these patients, CP could not completely be prevented. Placental abruption consists of two types by external bleeding: concealed abruption and revealed abruption [12]. The perinatal outcome of concealed abruption is worse than that of the revealed type [12, 13]. DFM is one of the symptoms of concealed abruption [13]. The rapid aggravation associated with conceal abruption may result in the poor outcome.

As approximately half of the pregnant women who had DFM were considered to have some form of hypoxia, the cord factor may be associated with the hypoxia group. In a recent retrospective study from Norway [14], velamentous insertion (adjusted relative risk [aRR]: 2.06) and true knot (aRR: 1.54) were significant risk factors for CP even after adjusting for maternal age and childbirth history [14]. Patients with CP who were complicated with cord‐related factor accounted for 15% [4]. Approximately 20% of CP with hypoxia cases involved marginal and velamentous insertion in our study. Although not mentioned in the Norway’s study, we showed that the degree of metabolic acidosis differed by the site of cord insertion. Compared with CP patients with normal site insertions, umbilical artery pH and base excess were better in those with marginal or velamentous insertion. Indeed, the median umbilical artery pH and base excess were 7.35 and −2.15, respectively, which were normal values. These findings suggest that temporary interruption of blood flow led to irreversible brain damage in patients with CP with marginal or velamentous insertion. However, upon blood flow restoration, the acid–base balance of umbilical blood returned to normal, resulting in a normal acid–base balance in the umbilical artery. Experimental animal studies demonstrated that temporary occlusion of umbilical blood flow can induce white matter injury [15]. It disrupts the normal progression of developmental myelination, inducing CP [16]. The reason why the hypoxia cases in the cord insertion group had lower pH values is unclear. This may be due to differences in the mechanisms leading to CP. A comprehensive evaluation, including detailed pathologic examination of the umbilical cord and placenta as well as thorough assessment of maternal factors such as thrombophilia, is needed to clarify these underlying causes. Putting aside, antenatal screening for cord insertion is important. If a fetus is found with a velamentous or marginal cord insertion, CP risk may increase. In addition, even if the umbilical artery pH is normal at delivery, the follow‐up of psychomotor development on infants with a velamentous or marginal cord insertion may be also necessary.

In this study, we roughly focused on DFM, and we could not examine qualitative or temporal changes of DFM. Recent several studies investigated the detailed change of the fetal movement. A case (fetal death, *n* = 164)–control (*n* = 569) study has shown that altered evening movement patterns or a sudden isolated episode of excessive movements may indicate fetal compromise [17]. Recent reviews have also highlighted that movement changes (e.g., increase) not only reductions warrant clinical attention [18, 19]. A case report showed that increased fetal movement decrease may suggest intrauterine fetal death [20]. Because our dataset did not include information on these qualitative features, we were unable to evaluate their contribution. Detailed fetal movement phenotyping is needed to better elucidate these relationships.

There are several limitations to this study. First, we did not compare patients without CP. The absence of a control group of patients without CP makes it impossible to compare with CP cases that had DFM. On the other hand, we could not compare CP cases with DFM and without DFM due to economic and ethical constraints. Second, further perinatal information could not be obtained due to confidentiality. Basic information including maternal age, height, weight, fetal sex, or maternal detailed complication was not included in the database, leading to incomplete analyses. Third, considerable interindividual differences in maternal perception exist, representing an inherent limitation in studies that rely on maternally perceived fetal movements.

## 5. Conclusion

DFM was seen in 8% of patients with CP. The duration from the DFMs to delivery was longer in the FMH group than in the placental abruption group. Fetuses with a velamentous or marginal cord insertion may have a risk of CP.

## Funding

No funding was received for this manuscript.

## Ethics Statement

This study was approved by the Institutional Review Board of our institution (approval number: rindai 21‐023) and the Review Board of JOCSC (approval number: rinjinsho 2021‐02). We did not obtain written informed consent from participating women. Because of the retrospective design, the ethics committee did not require informed consent from each patient. Information on how to opt out if desired was shown on a freely accessible website (https://www.sanka-hp.jcqhc.or.jp/documents/study_notice/index.html). The study conforms to the provisions of the Declaration of Helsinki. We did not use animals in this study.

## Consent

We did not obtain written informed consent from participating women. Because of the retrospective design, the ethics committee did not require informed consent from each patient.

## Conflicts of Interest

The authors declare no conflicts of interest.

## Data Availability

The basic data that support the findings of this study are available from the Japan Obstetric Compensation System for Cerebral Palsy. Restrictions apply to the availability of these data, which were used under license for this study. Data are available at https://www.sanka-hp.jcqhc.or.jp with the permission of the Japan Obstetric Compensation System for Cerebral Palsy.
